# Sensing the Arctic: Situational awareness and the future of northern security

**DOI:** 10.1177/00207020211048424

**Published:** 2021-09-29

**Authors:** Benjamin T. Johnson

**Affiliations:** Department of Politics, 7991 York University, Toronto, ON, Canada

**Keywords:** Arctic, Canada, surveillance, security, technology, situational awareness

## Abstract

This article considers the role of surveillance within security concerns related to the Arctic in Canada and North America. More pointedly, it examines how surveillance contributes towards situational awareness and the current emphasis on technological research and development to meet current and future security requirements. The article argues that Canada’s focus on surveillance within the Arctic offers a flexible strategy that navigates the complex and evolving security environment in addition to the political and fiscal realities of our time. However, the article warns that emphasizing the role of novel technology within strategic considerations risks undermining sound policymaking as the potential for new technology to transform defensive capabilities remains speculative. The article illustrates this approach to security by analyzing Canada’s Arctic surveillance capabilities and goals under the All Domain Situational Awareness (ADSA) program. Further, it links Canada’s efforts to North American defence by theoretically examining the role of surveillance in the Strategic Homeland Integrated Ecosystem for Layered Defence (SHIELD) concept and the recent NORAD/USNORTHCOM strategic outlook.

The Arctic has returned as a regional focus for Canada with increasing saliency in public discourse, mainly because climate change may transform the North into a strategic zone and potential battlespace over resources and transport routes.^1^ While there has been a surge in the literature on Arctic security, there has been minimal effort to capture Canada’s current Arctic policy relating to the state’s focus on developing its surveillance capacity. Like other states, Canada is bolstering investments and directing its efforts towards enhancing situational awareness in the Arctic by focusing on technological research and development.

Surveillance capacity and the enhancement of situational awareness are critical yet under-scrutinized aspects of Canada’s broader Arctic defence strategy. This article begins attending to this gap by demonstrating how a focus on enhancing surveillance offers a coherent pathway to meeting Canada’s security requirements presently and in the future. The state’s focus on surveillance capacity can achieve this in three principal ways. First, surveillance technologies and practices simultaneously occupy multiple capacities and disciplinary categories, specifically within scientific, security, and military fields (conventionally understood as their “dual use” character). The ability of surveillance technologies to encompass multiple capacities is necessary because Canada recognizes that several types of threats pose a danger to the Arctic and its communities outside of narrow military considerations. Second, the ability of surveillance technology to perform tasks outside of military practices contributes towards a holistic approach to security by facilitating a system-of-systems, or more starkly, an ecosystem model of defence. While a system-of-systems defence model involves the pooling of dedicated resources (such as sensors) into a complex network of interoperable components that amplifies the performance of the system as a whole, an ecosystem model theoretically goes beyond this and expands the scale of interoperable and integrated components to those outside of dedicated systems. Lastly, surveillance favours several practical considerations concerning financial and political constraints. Financially, the dual-use character of surveillance technology supports the Canadian government’s whole-of-government (WoG) approach to the Arctic through burden-sharing. Politically, surveillance technologies and practices may be more palatable to the Canadian public than other cost-intensive efforts or those with a weaponized character. Surveillance technology may also reduce the risk of provoking escalation through military spiral when those technologies remain within a defensive posture.

There has been a long history of research and development geared towards surveillance efforts in the Canadian Arctic, particularly during the Cold War. More recently, several surveillance-based projects have been put into motion within, and peripheral to, security concerns. For example, there is a great deal of sensor architecture in place that monitors environmental phenomena, including ice floes and atmospheric disturbances. Canada’s Department of National Defence (DND) has deemed these civilian-based sensors important for inclusion based on security considerations. More evidently, there are many research and development efforts currently underway exploring both evolutionary and revolutionary technologies for Arctic surveillance, some of which may contribute to North American Aerospace Defense Command (NORAD)’s modernization. The current focus on NORAD modernization has posited the need for an ecosystem approach to surveillance and all domain awareness, which conceptually involves integrating multiple sensor systems and data streams (within and outside of the Arctic; potentially global in orientation) into a cohesive intelligence picture. Accordingly, this intelligence picture should enable pre-emptive action through predictive analysis of the future. However, this article examines the concept of ecosystem surveillance through a critical lens by tempering the expectations of technology to radically transform defensive capabilities given the degree of uncertainty inherent to technological development and use.

This article proceeds as follows. The next section begins with a review of Canada’s earlier efforts to develop surveillance technologies while emphasizing elements of continuity between Stephen Harper’s Conservative and Justin Trudeau’s Liberal governments through their mutual interest in technological development for Arctic situational awareness. The article then examines recent surveillance efforts, particularly concerning environmental sensing, and links those efforts to the ongoing research and development projects led by the DND, specifically the All Domain Situational Awareness (ADSA) program. The article then shifts to engage with current discussions surrounding the modernization of NORAD and Canada’s potential contribution by focusing on enhanced Arctic surveillance requirements. Lastly, the article concludes with a brief review of its argument concerning the need to be wary of overly deterministic accounts of how technology can and will revolutionize defence capabilities.

## A history of surveillance

Like the conceptual evolution of security’s expansion beyond narrow military considerations following the end of the Cold War, Canada has adopted a holistic understanding of security to encompass various issues that threaten the Arctic and its communities, including, but not limited to, military threats.^2^ This breadth of issues stems from the intersection of several trends globally, including climate change; the evolution in, and proliferation of, technology; and shifts in the relative power among states and non-state actors.^3^ Canada’s widened security perspective is also related to the Arctic’s political, geographic, and environmental characteristics, which historically have contributed to Canada’s sovereignty concerns fuelling the need for increased surveillance capabilities.^4^ Surveillance represents a complex set of practices that have “over perhaps the past 40 years [...] emerged as the dominant organizing practice of late modernity.”^5^ Within security and defence considerations, surveillance broadly refers to “the systematic observation of aerospace, cyberspace, surface, or subsurface areas, places, persons, or things by visual, aural, electronic, photographic, or other means.”^6^ At a basic and instrumental level, surveillance serves as a performative tactic in fulfilling the need for situational awareness, a key concern for Canada, given the Arctic’s large and diverse landscape. The Arctic represents approximately 40 percent of Canada’s total geographic land area and 162,000 kilometres of coastline, the largest in the world. In line with the conceptual diversification of security within Canada’s Arctic policy framework, Canada has prioritized a WoG approach to Arctic defence and security. This approach includes efforts related to developing Canada’s Arctic surveillance capacity, enabling burden-sharing in cost, development, and implementation. The ability to provide situational awareness theoretically imbues the state with an exclusive power of perception over the physical and electromagnetic domains. Situational awareness includes, but is not limited to, surveillance, which enables the production and dissemination of intelligence through practices of *sensing* that can ultimately inform decision-making for policymakers and field commanders.^7^

Within intelligence, surveillance, and reconnaissance capabilities (ISR), sensing refers to the joint practices of surveillance and intelligence (see Table 1). These two practices are applied across the spatial and temporal vectors, meaning that situational awareness requires a diverse suite of sensors that provide historical and persistent data feeds to create actionable intelligence. Across the spatial frontier, geography is illuminated through surveillance technologies using the visible and non-visible spectrum (such as infrared). The surveillance of environments and objects is systematically practiced over time to identify trends. Intelligence practices are used to make sense of raw spatial data by correlating events and objects within and across environments using these historical and persistent data feeds. These correlations can then be used to make predictions about future events by identifying the “patterns of life” of actors through their behaviour. In terms of security, using intelligence practices to make predictions enables the creation of “heat maps” by directing attention to areas where threats exist or are likely to emerge using risk-identification markers to facilitate resource allocation and decision-making.

**Table 1.** Sensing for dynamic situational awareness.

*Sensing*	Spatial	Temporal
Surveillance	Illumination (visible, non-visible) of environments, objects	Historical data, persistent coverage
Intelligence	Data on events, objects, making correlations	“Patterns of life” analysis, predicting behaviour, generating “heat maps”

For the modern context, sensing requires a fluid combination of surveillance information with other data sets and the ability to translate that data assemblage into a coherent intelligence picture suitable for human decision-making. Surveillance technology is especially appealing through its dual-use capabilities, which involve applying technology to civilian and military goals. Additionally, technological development possesses an economic dimension in which these technologies are both fiscally conservative and economically productive in their own right through the procurement of Canadian businesses and expertise. Modern surveillance technologies—including satellites, underwater sensors, drones, radar, among other platforms—enjoy more potential in terms of their productivity relative to the development of militarized systems exclusively and may be more acceptable to a domestic audience. Perhaps more importantly, surveillance technologies theoretically allow the state to perform security remotely, digitally, and minimally across a spectrum of requirements while reducing the risk of provoking escalation.^8^

Rhetorically, contemporary discourse often frames the Arctic as a future conflict space, supporting the need for greater attention and resources. A great deal of the thematic content propelling the dramatic side of Arctic security discourse considers the renewed tensions between Russia and western states stemming from Russia’s turn towards a militarized foreign policy in the Arctic and elsewhere in the world.^9^ The “new Cold War” imagery flooding Canadian media began earnestly in 2007 as Russia planted its national flag on the North Pole seabed, which had no legal implications for possession of any Arctic territory. Russia’s symbolic claim to the Arctic as *terra nullius* may be juxtaposed against the Harper government’s response, which included a great deal of “hard” political rhetoric that projected a need for, and investment in, military ships, infrastructure, and capabilities that would “protect” Canada’s northern sovereignty.^10^ However, time has revealed a significant disconnect between Harper’s rhetoric and policy as actual investment in Arctic capabilities, and the completion of projects during that era do not match the glut of ventures announced at the time.^11^

Emblematic of a less blatant security framework predicated on military show and strategy, the Trudeau Liberals have signalled at least a symbolic shift from the Harper Conservatives, moving away from Harper’s hardline discourse while emphasizing the region as a space for cooperation and stewardship.^12^ Efforts by the Trudeau government to portray a cooperative image amenable to Arctic governance also sync with broader regional forces that characterize the Arctic as a space dominated by the “normal” interstate politics of cooperation, which are pursued through institutions and governance regimes.^13^ This shift is not unusual or altogether new, as Canada’s Arctic strategy has routinely shifted in strength and emphasis in national and foreign policy over time.^14^ Indeed, the move towards the language of cooperation is representative of Canada’s foreign policy support for liberal internationalism as a whole since the end of World War II.^15^ Additionally, there has been a clear pivot towards a more inclusive role for NATO in Arctic defence matters, which stands in contrast to earlier efforts by Canada during the Harper era to resist such inclusion. Harper’s reluctance was due to Canada’s prioritization of narrow domestic interests and a concern that an official role for NATO in the Arctic could incite a reactionary move by Russia.^16^

Despite the apparent rhetorical change, there is a great deal of symmetry between the Harper and Trudeau eras,^17^ particularly in terms of the state’s focus on research and development related to Arctic surveillance technologies. Canada has repeatedly articulated a need for greater surveillance capacity to fulfill the state’s security requirements and demonstrate authority.^18^ Canada’s surveillance concerns are also linked to continental defence requirements embodied by the Distant Early Warning Line (DEW) and its upgrade, the North Warning System (NWS).^19^ The technical and instrumental logic underpinning research and development efforts related to enhancing Canada’s Arctic surveillance capacity also demonstrates a longer-term thematic consistency, perhaps most notably with efforts pursued during the Cold War. For example, given the importance of Soviet submarines and their nuclear component, western naval strategy focused on the Greenland-Iceland-UK (GIUK) gap, where surveillance practices became a central part of that strategy. Additionally, the United States led the development of a chain of underwater listening posts known as the Arctic Sound Surveillance System (SOSUS) and combined that development with acoustic research in the Kara and Greenland Seas areas.^20^ SOSUS represents to Gary Weir “[t]he most ambitious and effective project undertaken during the Cold War next to the hydrogen bomb,” and directly informed a joint study between Canada and the US examining the feasibility of developing a passive-sonar system sometime in the 1980s. This system was designed to monitor subsurface movement in the Canadian Arctic as Russian submarines became quieter and more advanced.^21^

Following the end of the Cold War and the 11 September 2001 attacks, the DND continued to pursue science and technology (S&T) initiatives related to Arctic surveillance in the early 2000s, the most notable of which were the Pacific Littoral ISR Experiment (PLIX) and its follow up, the Littoral Intelligence, Surveillance, and Reconnaissance Experiment (ALIX). As a whole, these two projects were designed to contribute to an integrated ISR architecture, consisting of a sensor and communications network that would be linked to decision-makers and commanders within a battlefield environment.^22^ More recently, Defence Research and Development Canada (DRDC) released its report on the Canadian Arctic Underwater Sentinel Experiment (CAUSE), undertaken at the chokepoint of Gascoyne Inlet in the summer of 2017. CAUSE was part of DRDC’s larger five-year project titled the Northern Watch Technology Demonstration Project (NWTDP). The NWTDP was concerned with developing and installing various sensor technologies in the Arctic around Gascoyne Inlet, which served as the site for Canada’s earlier Cold War prototype detection system due to the Inlet’s function as a natural chokepoint for ships and submarines into the region. In turn, the objectives of CAUSE were to see if the acoustic arrays deployed as part of the NWTDP in the summer of 2015 were working while conducting various technological tests related to surveillance and measurement.^23^

## Ecosystem surveillance

Current surveillance efforts embody a developmental logic similar to that of these earlier projects, especially in terms of their functional capacity to operate within a networked architecture, which emphasizes cost-benefit analysis and open-source data integration with primary surveillance data. Canada’s whole-of-government approach to the Arctic explicitly deploys this developmental logic within the state’s Arctic surveillance and security efforts, which relates to a spectrum of concerns outside of narrow defence interests. Given the range of security threats presented to the Arctic combined with Canada’s extensive territory and the logistical issues confronting state governance, a WoG approach supports burden-sharing and the flexible application of authority between multiple government departments, communities, and other actors, at least in principle.^24^ For instance, environmental dynamics are a crucial issue for security interests in the Arctic. Just as weather stations provided important security information for both Canada and the United States from the early twentieth century onward,^25^ there is a great deal of current interest in advancing Canada’s scientific surveillance capacity to create more accurate prediction models of daily environmental patterns and the Arctic’s broader ecological transformation through climate change. One of the most prominent examples is the International Polar Years (IPY), in which multiple nations coordinate their polar expeditions and scientific research for at least a year. Within this program, and of particular interest to defence personnel, is the Year of Polar Prediction (YOPP) component, one part of the larger Polar Prediction Project (PPP). The YOPP is composed of a network of natural scientists from the World Meteorological Association (WMA), the World Weather Research Programme (WWRP), and the World Climate Research Programme (WCRP), along with a host of research centres, universities, and other institutions researching earth-based weather in the Arctic.

**Figure 1.**
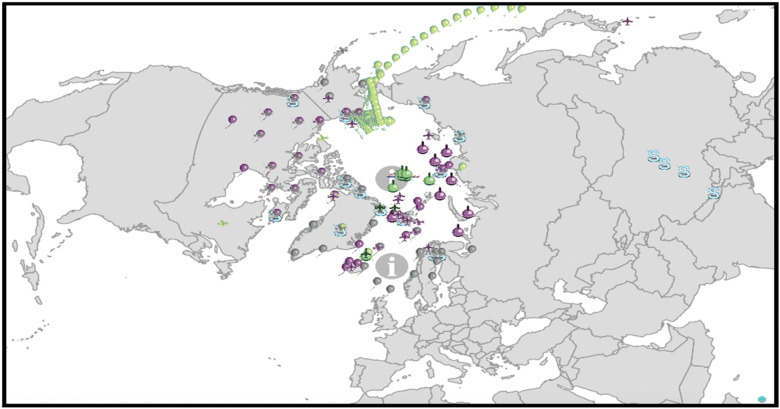
Sensors that are existing and under continuous activity; those planned or under consideration, including airborne, buoys, automatic weather stations (AWS), radiosondes, other, and supersite.^63^

The Arctic is often framed as a region of low infrastructural development and as retaining a frontier quality. However, there has been less appreciation for how developed the region is in environmental and ecological surveillance coverage (see Figure 1). While the observation layer of sensors and other surveillance platforms collated by the YOPP may appear extraneous to security concerns, environmental awareness and predictive capabilities support several governance efforts, including search and rescue (SAR) operations and other human activity in the Arctic.^26^ As the government of Canada states, “many safety, security and defence efforts in the Arctic and the North are reliant on sound weather, water, ice, and climate information, alerting and warning services to help mitigate operational risks.”^27^

**Figure 2.**
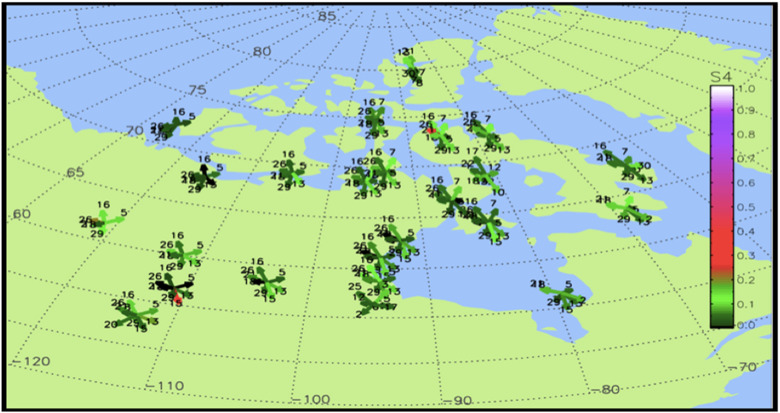
CHAIN real-time scintillation map; date captured: 15 May 2020.^64^

Likewise, the role of space weather is monitored by the Canadian High Arctic Ionospheric Network (CHAIN) and has been identified as a critical issue of practical concern for the DND as ionospheric disturbances can affect network communications and global positioning systems (GPS).^28^ Specifically, ionospheric scintillation in the Arctic can affect GPS and communications networks within C4I systems (command, control, communication, computers, intelligence). Consequently, modern defence efforts require a robust understanding of the solar-terrestrial system and scintillation patterns (see Figure 2). As a whole, polar environmental research supports security and military operations because these operations will increasingly depend upon in-depth knowledge of the Arctic’s unique environment to be successful.^29^

**Figure 3.**
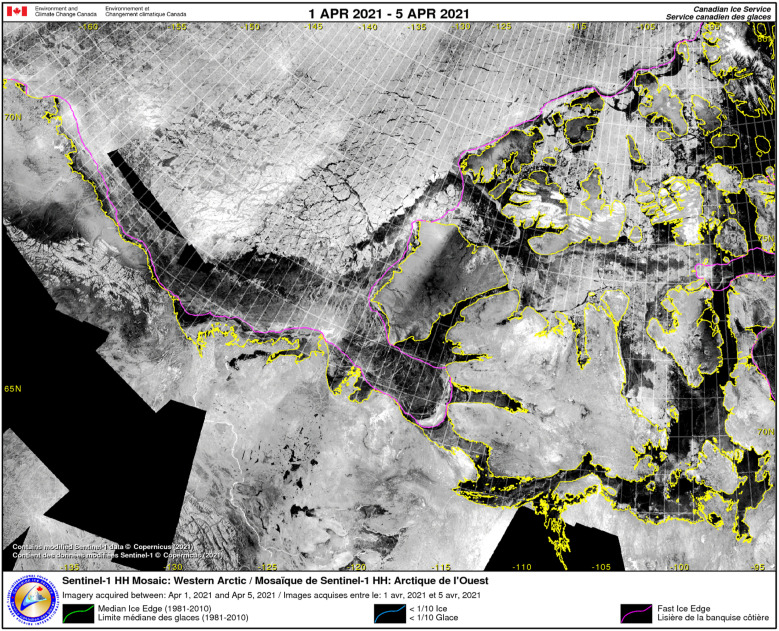
RADARSAT Mosaic Western Arctic; imagery acquired between 1 April and 5 April 2021 (at least partially composed of Sentinel-1 data due to the transition from RADARSAT-II to RCM).^65^

In addition to the YOPP and CHAIN, Canada is pursuing several other surveillance-related efforts in the Arctic. Emblematic of these efforts is the Inuit Guardians Program, which includes projects that bridge Indigenous knowledge and experiences with modern technologies to monitor environmental trends and their effects on resources (such as fish and caribou) and Indigenous lands.^30^ Transport Canada’s Ocean Protection Plan also provides environmental monitoring*,* which involves a $1.5 billion investment towards developing a marine safety system, including the ability to provide real-time awareness of environmental events (e.g., oil spills), emergencies, and marine traffic.^31^ Canada expects these requirements to grow as the flow of Arctic traffic increases and melting sea ice creates additional safety issues for marine navigation, requiring a real-time awareness of daily ice floes. RADARSAT Constellation Mission (RCM), the most recent RADARSAT satellite developed by MDA Inc. (formerly MacDonald, Dettwiler and Associates), provides this awareness (see Figure 3).

In contrast to its predecessor, RADARSAT-II (still in operation and owned by MDA Inc.), RCM, and all of its sensor data are owned by the Government of Canada, while MDA served as the developer. This transfer of control is indicative of the federal government’s efforts to consolidate its power over technologies and information that it understands to be essential for supporting the national interest and security.^32^ In addition to RCM’s various surveillance functions, the satellite is equipped with an automatic identification system (AIS) that can combine ship data with RCM surveillance and open-source data. RCM’s AIS capabilities support the Canadian Coast Guard’s own AIS infrastructure across the Canadian coastline and its long range identification and tracking (LRIT) capabilities provided by the Iridium satellite network for Arctic operating vessels. Importantly, Fisheries and Oceans Canada distributes AIS and LRIT data to other government departments interested in national security.^33^

Arctic surveillance is also supported by the human element, as embodied by the Canadian Rangers. Historically, Ottawa considered the Rangers a valuable asset in contributing to ground surveillance efforts in the Arctic while supporting the state’s legal claims in the region.^34^ Current discussions on implementing the priorities outlined in Canada’s defence white paper, *Strong, Secure, Engaged,* indicate continuity with these historical efforts and position the Canadian Rangers in a supportive role within strategic efforts to monitor and defend the Arctic.^35^ Specifically, the Rangers are expected to “[a]ugment regional maritime domain awareness” by relaying “what is and what is not normal in their local areas, fostering a ‘see something, say something culture’” and embodying their motto of *Vigilans* (“The Watchers”).^36^

Surveillance thus already represents a fundamental component of Canada’s governance strategy in the Arctic and is a widely shared practice across multiple departments. Technology also enjoys a clear focus as a critical tool for enhancing the state’s surveillance capacity and providing situational awareness. Anticipating the government’s future need, Canada is pursuing several research and development initiatives related to building advanced sensing technologies. The ADSA program is one such avenue of development and is also expected to contribute to Canada’s modernization efforts in NORAD and continental defence.

Having established the role of technology in Canada’s surveillance efforts, this article now considers research and development initiatives related to advancing Arctic surveillance capacity in the future.

## All domain awareness and North American defence

The ADSA program exemplifies Canada’s experimental efforts to develop surveillance technology in the Arctic. The program is a broader funding initiative with private and public partners that was launched in 2015 and has focused on developing Canada’s surveillance capacity in the Arctic across a spectrum of technologies in order “to produce innovative solutions to surveillance challenges in the North.”^37^ Under the ADSA project, Public Works Canada issued a call for proposals within two broad streams in 2016 that sought solutions for “Air, Surface, and Sub-Surface Surveillance, and Sensor/Information Mixes S&T” as well as “projects that improve understanding of critical infrastructure (CI) vulnerabilities.”^38^ The ADSA program focuses on assessing and developing surveillance technologies for the future, especially those that can automatically detect and classify objects of interest (including advanced weapons systems) within complex and densely populated environmental conditions. There is currently a significant gap in the state’s identification and parsing abilities, indicating the need to classify and discriminate “threatening” from “non-threatening” objects in the Arctic’s challenging environmental conditions and diverse landscape. This requirement is expected to increase relative to the future growth in Arctic sea vessel traffic. Specifically, greater access to the Arctic resulting from extended ice-free periods will create a set of vulnerabilities in the border regions of littoral Arctic states. Consequently, one of Canada’s chief concerns in the Arctic is being able to secure the “flow of goods and people at ports of entry”^39^ and monitor the region’s extensive coastline approaches.

Enhancing Canada’s Arctic situational awareness capacity is important beyond domestic considerations and is critical to North American defence more widely. Specifically, Canada’s research and development (R&D) efforts for enhanced surveillance and intelligence capabilities within the ADSA and other related programs are also expected to contribute to NORAD’s modernization and renewal of the NWS.^40^ For example, Canada is interested in the practice of quantum illumination and researching its potential application with radar systems. Quantum is poised to become a significant avenue of research focus for Canada with the recent release of National Defence’s Quantum Science and Technology Strategy. Regarding surveillance, there is a research focus on “quantum sensing,” which surpasses the performance of traditional applications in terms of measurement accuracy.^41^ In addition to the Quantum S&T Strategy, the University of Waterloo’s Institute for Quantum Computing (IQC) received a $2.7 million contract through the ADSA program. Under this research contract, technology and techniques enabling quantum illumination are being studied for their potential contribution to remote sensing methods that overcome the natural environmental challenges posed to conventional radar presented in the Arctic.^42^

NORAD’s modernization requirements have generated a great deal of discussion in recent years, given the need to keep pace with advancing capabilities by competing states, including those related to cruise missiles and hypersonic weapons. NORAD is a binational organization between Canada and the United States, and its mission is focused on aerospace warning and control for North America, with the maritime approach added to its mission suite in 2006. Historically, NORAD emerged from an instrumental need for the United States to create a spatial buffer against Soviet bombers during the Cold War, which required joint efforts with Canada to build a credible defensive posture given the indivisibility of airspace.^43^ The early detection of Soviet bombers was enabled through radar surveillance, including the DEW line. NORAD persisted as an institution following the end of the Cold War, and shifted its attention after the 11 September 2001 terrorist attacks to focus on defending North America from airborne threats that emerged within and outside of national airspace. Current warning capabilities are provided through the “detection and validation” of threats using “a central collection and coordination facility for a worldwide system of sensors” collated at NORAD and US Northern Command (USNORTHCOM)’s central facility at Peterson Air Force Base, Colorado.^44^ NORAD’s sensor suite includes the current NWS, which evolved out of the earlier DEW line in the late 1980s and consists of fifty-four long- and short-range radars in the Arctic (forty-seven of which are located in Canada) that form a “tripwire” stretching from Labrador to Alaska. Critically, the NWS is reaching the end of its lifespan and requires upgrading to serve as an effective deterrent to near-peer competitor states (namely Russia and China), who are developing advanced weapons and delivery technologies.

Discussions centred on NORAD’s modernization indicate the potential for the organization’s next evolutionary step, where surveillance continues to factor heavily into its overarching mission goals. Advances in weapons delivery technologies that are rendering current surveillance capabilities obsolete, combined with the greater interest in the Arctic for economic exploitation, require improved early warning capabilities in the region to protect North America’s security and strategic advantage shaped by distance. While the Arctic has remained a focus within Canadian policy since the end of the Cold War to greater and lesser degrees, the United States has only recently pivoted towards a renewed interest in the region for supporting national security.^45^ Thus, given this current binational focus, technological research and development for enhanced surveillance capabilities is a significant policy area directed at meeting Arctic security requirements. Research and development efforts potentially represent a crucial avenue of contribution by Canada towards Arctic surveillance in service of both national and binational security. Besides quantum-based R&D, other ADSA projects may aid the modernization of NORAD and facilitate greater sensor and intelligence integration with the United States while contributing towards the minimum 20 percent R&D spending requirement within NATO.

A significant degree of integration will be necessary to achieve the level of technological readiness required for dynamic situational awareness in the Arctic for continental defence. These needs are conceptually embodied by the Strategic Homeland Integrated Ecosystem for Layered Defense (SHIELD) framework advanced by former NORAD Commander General Terrence J. O’Shaughnessy and the current NORAD and USNORTHCOM strategy led by Commander General Glen VanHerck.^46^ Both SHIELD and NORAD/USNORTHCOM strategy share an emphasis on enhancing surveillance and intelligence capabilities and point to how sensing is critical for providing complete situational awareness in the Arctic. While distance was technologically reduced as a strategic buffer long ago, there has been a radical transformation in the logic of distance by advances in conventional weapons systems. Specifically, developments in offensive capabilities by peer competitors have focused on closing the spatial gap afforded to North America through conventional means by developing weapons that remain within the threshold of use (below nuclear) as new technology reduces the problems of time, space, and detection. Consequently, these technological advances represent a major problem for current NORAD surveillance and warning capabilities, creating a significant security gap in the Arctic and North America.

The current National Defense Strategy (NDS) of the United States signals a clear thematic departure from its previous focus on countering extremism to re-engaging with its near-peer states within a framework of strategic competition.^47^ However, there is a great deal of consistency between the post-9/11 era and the current US focus on interstate strategic competition concerning the role of surveillance technology in security practices. Notably, both counterterrorism efforts and the current international security environment are understood to be rooted in complexity and relationality on a global scale. Put otherwise, the rapid evolution of technology, its proliferation among state and non-state actors, combined with other trends at the international scale (including climate change, competitive behaviour by Russia and China, societal instability in multiple contexts, among others) is creating a strategic environment that is increasingly difficult to assess and navigate. This difficulty is borne out because none of these issues exist or operate discreetly, creating a complex web of constantly shifting threats. Hence, this complexity creates operational challenges for analysis, resource allocation, and decision-making for security practitioners, particularly when considering future requirements.

Conceptually, SHIELD and the current NORAD/USNORTHCOM strategic vision follow US doctrine and respond to this increasingly complex environment. The focus on surveillance and intelligence represents something of a strategic evolution of the United States’ earlier notion of achieving “full-spectrum dominance,” which involved complete control over a battle environment’s physical and electromagnetic domains through technological superiority. In contrast, SHIELD, NORAD/USNORTHCOM, and current US strategic doctrine extend the logic of full-spectrum dominance much more acutely into the sensing sphere to achieve something akin to a full-*spectral* dominance. More pointedly, technologically mediated surveillance practices may exploit the complete range of visible/non-visible wavelengths and auditory frequencies to enable real-time all domain situational awareness. Nancy Teeple and Ryan Dean capture this evolution in their characterization of SHIELD as involving the fusion of sensors and data from multiple sources “into a comprehensive picture that identifies threats at the extreme edge of awareness.”^48^ This “edge of awareness” involves both a spatial and temporal dimension, where sensing conceptually links to the expansion of state power into the ether of threat activity, beyond a “system-of-systems” approach and into a complete “ecosystem” of sensor architectures within a “global sensing grid.”^49^ Likewise, the current NORAD/USNORTHCOM strategic doctrine prioritizes all domain awareness within a global framework to support information dominance that can be mobilized towards rapid and flexible action.^50^ In terms of sensing, the “edge of awareness” points towards the blurring of space and time as the threat environment is increasingly complex and populated by actors that are or will be able to circumvent the geostrategic benefits once enamoured to North America.

For efforts directed at modernizing NORAD to detect and counter these weapons in addition to the proliferation of other security concerns, this complex threat environment indicates the need for advanced surveillance capabilities to build effective deterrence by denial.^51^ More fundamentally, any modernization of NORAD requires policymakers to rethink its defensive posturing and the defence/offence strategic framework as a whole because defeating an attack from an advanced weapons system necessarily involves detecting and potentially defeating that attack “from birth.” Traditionally, NORAD’s mission emphasized a defensive posture through its warning and response capabilities against airborne threats to North America. However, current strategic thinking points to the need to develop a *pre-emptive* offensive capacity to create a credible deterrence against current and future capabilities and reconstruct North America’s spatial buffer.

Indeed, pre-emption represents the starkest theoretical diversion from earlier surveillance efforts because pre-emption (or, as General O’Shaughnessy calls it, “predictive analysis”) involves thinking relationally rather than linearly within a global space as a futurized construct. In O’Shaughnessy’s words, we need to be able to make decisions that “are thinking about two or three moves downstream.”^52^ The extensive technologies related to artificial intelligence (AI) (machine vision, deep learning, and other applications) are expected to play a significant role in NORAD’s future imaging and intelligence regime.^53^ This strategic outlook indicates that AI will be required to automatically examine multiple surveillance nodes across the globe and translate them using predictive analysis into an intelligence picture suitable for decision-making needed to pre-emptively shape the battle environment.^54^ The growth of AI-based sensor platforms across the Arctic represents a key area of potential contribution for Canada within NORAD modernization efforts. In principle, the integration of speed through the dispersion of agency across several discrete but networked actors is designed to mirror other “system-of-systems” approaches, such as within decentralized economic production networks that enable redundancy and adaptability. Developments in Arctic security technologies demonstrate a particular resonance with these broader trends, especially in state-led efforts to integrate surveillance data with other data sources. Moreover, temporally compressing analysis cycles produces a “just in time” model for intelligence dissemination and decision-making.^55^ In principle, the use of AI for these purposes resembles other autonomous behaviour developments inspired by biological neural networks that perform complex cognitive tasks, including those that overwhelm human ability. The sum result of these efforts is expected to “contribute to joint efforts between Canada and the United States to improve surveillance capabilities in support of Canadian and NORAD requirements and missions.”^56^

To summarize, the international sphere within the current era is defined by the intersection of several trends, including advances in technology, the return of strategic competition, and the proliferation of non-state actors such as terrorists and criminals. The Arctic is a critical theatre within this complex environment for two key reasons. First, climate change enables or will enable greater access to the Arctic, so there is, or there is predicted to be, a surge in interest by state and non-state actors to access the Arctic for their own strategic and economic benefits. Second, like the Cold War environment, the Arctic is a strategic buffer zone for North American defence, as an attack on southern targets in Canada and the United States is likely to travel through the Arctic. As the Arctic opens up due to warming global temperatures and extended ice-free periods, this will increase North America’s vulnerability to the proliferation of threats resulting from these broader international trends. This vulnerability is particularly important for Canada given the Arctic’s size, its extensive coastline, and a relative lack of northern development in terms of infrastructure. Thus, enhancing surveillance and intelligence capabilities through technological superiority in the region has become a central strategic goal for Canada, which shares this logic with NORAD’s current strategic outlook and US defence policy more broadly.

## The politics of surveillance

We must be wary of arguments fetishistic of technology and purely technological solutions to current security challenges in the Arctic and North America, especially those emphasizing the importance of revolutionary development. For instance, the SHIELD concept is an ideal-type architecture presented as an instrumental requirement to the structural forces of current trends in the security environment rather than an existing defence grid. From Reagan’s Strategic Defense Initiative (nicknamed “Star Wars”) to the Revolution in Military Affairs (RMA), recent history demonstrates that creating a defence strategy centred on the development of technology does not mean such strategies are feasible in the real world or that they automatically produce desired outcomes, especially given considerations of time and money. Even the most sophisticated technology in existence can defy expectations of use. Technology may indeed be transformative of warfare (as it has been repeatedly). However, it has never done so independently of conflict’s political and social reality, something Carl von Clausewitz noted long ago. We do not have to look much further than the recent experiences of the US-led war on terror. As the drawn-out engagements demonstrated, the much-vaunted geostrategic advantage provided by technological dominance did not help US forces anticipate the complications that arose in Afghanistan and Iraq.

The strategic prioritization of all domain awareness and information superiority is equally a political orientation to the world as it is a technological goal to achieve sensing dominance. Therefore, achieving a globally integrated all domain awareness involves many questions and issues, including but not limited to actual technical capacity. Andrea Charron points to these issues by noting that shifting NORAD into an offensive command posture may not be acceptable to the Canadian public, especially within the fiscal and political environment created by the COVID-19 pandemic.^57^ There are several additional considerations for Canada to make advanced sensing capabilities within NORAD a reality. Beyond the technical concerns, there are multiple issues related to intelligence dissemination and ownership and questions concerning Canada’s role in shaping an updated NORAD to define the institution’s offensive capabilities.^58^ Most importantly, Charron is wary of prioritizing automation over keeping humans “in the loop.” Specifically, Charron argues that “[o]n many occasions, however, disaster has been obverted because a soldier or analyst doubted what a computer screen was telling him/her or questioned the data blinking on their screen.”^59^ Charron’s critique indicates that we must be conscious not to overtly fetishize technology (both existing and experimental) by reducing our understanding of it to its instrumental function. This awareness is essential as the discrepancy between strategy and reality is often not revealed until using technology in theatre conditions. Rhetorically, technology is often treated as deterministic of outcomes in a linear pathway (i.e., if we possess *x* technology, outcome *y* will result). However, the outcomes functionally derived from notions of technological dominance are often divorced from how a technology ends up working (or not working). This fetishism is especially troublesome with experimental technologies like quantum because they are far from proven despite their potential for significant returns as a factor of investment. As Frank L. Smith III argues on the security hype of quantum, “It is uncertain whether quantum technologies will live up to these high expectations. If they fall short, they will not be alone. We interpret new and emerging technologies on the basis of collective expectations about imagined futures, including our dreams and nightmares. These expectations are often unmet following hyperbole or hype.”^60^

In light of these concerns, the federal government’s current surveillance strategy offers a sufficient degree of flexibility to meet Canada’s more immediate and future needs. This strategy may be broadly summarized as integrating existing sensors and data points with other “off the shelf” solutions while pursuing riskier research and development initiatives under the ADSA program and other efforts. There are several inherent benefits to this strategy that have political, economic, and strategic dimensions. Politically, surveillance practices and technology possess an aesthetic and normative character concerning their dual-use capacity that supports Canada’s security requirements as a whole while remaining on the fringe of an explicitly militarized capacity. Remaining outside of explicit militarization helps signal the state’s intention domestically and internationally, thereby aligning with broader efforts to preserve the Arctic as a regional space of cooperation. The dual-use capacity of surveillance technology also entails aspects of economic efficiency. Enhancing surveillance capacity in the Arctic can be primarily supported through “off the shelf” solutions, while investment in surveillance R&D remains conservative compared to other potential avenues of defence spending.^61^ Focusing on technology is also productive in its own right. The federal government has made efforts to showcase Canadian expertise in its surveillance domains on the international stage (especially in space-based surveillance) with a significant contribution by Canadian firms. Further, R&D spending can be enhanced where there is sufficient promise in a technology, which can continue to support Canadian economic development through procurement as the ADSA program has done. Canada’s research expertise is significant, especially in dedicated institutions focused on revolutionary technologies like quantum. Lastly, focusing on surveillance technology offers a strategic advantage relative to more path-dependent forms of development. While much effort has been made to predict the future security environment and futurized ways of war, these remain speculative endeavours. Thus, Canada’s focus on Arctic surveillance offers an adaptive pathway across the political, economic, and strategic frontier. Focusing on surveillance and enhancing situational awareness within the Arctic offers a clear pathway in the short term that can support medium- and long-term efforts in the Arctic and towards continental defence as these longer-term goals become better defined.^62^

However, this does not mean that difficult choices will not have to be made relatively soon, especially in the context of NORAD and missile defence. Additionally, despite these benefits, policymakers must be careful not to depoliticize surveillance as a practice or its associated technologies, given that their development involves many active political choices. Further, we must be conscious of how Canada’s security priorities may deviate from the US emphasis on peer competition and how domestic constraints may influence Canada, including public opinion and limited fiscal budgets for the foreseeable future. This environment may also affect Canada’s strategic approach to development. Limited budgets may incentivize a greater reliance on experimental and unproven technologies or a reliance on existing technologies that may quickly become outdated or circumvented. It remains to be seen how Canada will navigate its role as a middle power within the perceived return to great power politics in terms of its support for offensive capabilities within the Arctic and NORAD. However, for the time being, it appears that balancing Canada’s domestic interests with the priorities of continental defence and regional governance will require delicate navigation.^63^ The policy intersection of these scales is not necessarily symmetric, nor are they always compatible,^64^ as the promotion of one level may require compromise in the other.^65^

## Conclusion

This article has argued that Canada’s Arctic defence strategy focusing on surveillance and technological development is sensible from a policy standpoint because surveillance technologies and practices are both *defence*-oriented (in a narrow military sense) and *security*-oriented (in a broadened sense). Surveillance technologies possess a “dual-use” character that occupies multiple aesthetic categories. Additionally, dual-use technology possesses a common-sense appeal through its link to “protecting” sovereignty (as an inviolable right of *all* states) without risking provocation in a way that offensive development may lead towards a military spiral. Technological development for Arctic situational awareness is understood to be both fiscally conservative and economically productive in its own right. While often experimental, surveillance technologies are relatively cheaper than other forms of defence investment and support economic development by contracting Canadian firms and expertise. Lastly, surveillance has a practical character in that it lends itself well to a whole-of-government approach by distributing capacity and burden across multiple departments. This strategy enjoys the benefit of servicing Canada’s more immediate security needs in the Arctic while remaining sufficiently flexible to adapt to medium- and long-term goals as these technologies advance and Canada’s role in North American defence becomes clearer, including its possible contribution to missile defence.

Despite the pragmatic role that surveillance offers, policymakers must be wary of any deterministic accounts of how technology will save us from an increasingly threatening world. In particular, any role that sensor technology will play in the modernization of continental defence systems through NORAD must be considered within the broader political context outside of narrow instrumentalist reasoning derived from a technological focus. Remaining cognizant of technology’s political and social context is especially important concerning the strategic emphasis on all domain awareness, which prioritizes the development of a technological ecosystem with the potential for global reach by integrating numerous sensor networks and data sets. The operational logic of SHIELD and the current NORAD/USNORTHCOM strategy enjoys a broader symmetry with US strategic thinking. The strategic approach of these cases stresses the role of technology, especially artificial intelligence, in producing the material capabilities for dominating future conflicts through surveillance, information superiority, and decision cycle dominance within an all domain battlefield. Undoubtedly, current trends within the international sphere are worrisome and invite serious attention to the defence needs in the Arctic and North America. Whatever solutions are delivered in the coming years, an enhanced surveillance capacity supported by technological development will undoubtedly factor heavily into those measures. However, we should not be so bold as to assume that technology will automatically deliver the promised salvation that geostrategic thinking might suggest when divorced from the messy reality that any Arctic future may entail.

## Notes

1 See Rolf Tamnes and Kristine Offerdal, eds., *Geopolitics and Security in the Arctic: Regional Dynamics in a Global World* (London and New York: Routledge, 2014).

2 For an overview of how Arctic security is understood and analyzed across state-centric and critical orientations, see Gunhild Hoogensen Gjørv, Marc Lanteigne, and Horatio Sam-Aggrey, eds., *Routledge Handbook of Arctic Security* (New York: Routledge, 2020); Government of Canada, “Arctic and Northern policy framework: Safety, security, and defence chapter,” 10 September 2019, https://www.rcaanc-cirnac.gc.ca/eng/1562939617400/1562939658000 (accessed 13 April 2021). For a discussion of Canada’s Arctic and Northern Policy Framework, see Peter Kikkert and P. Whitney Lackenbauer, “Canada’s Arctic and Northern policy framework: A roadmap for the future?” *Arctic Yearbook 2019*, https://arcticyearbook.com/images/yearbook/2019/Briefing-Notes/9_AY2019_BN_Kikkert_Lackenbauer.pdf (accessed 17 April 2021).

3 National Defence, *Strong, Secure, Engaged: Canada’s Defence Policy*, Government of Canada, 2017, http://dgpaapp.forces.gc.ca/en/canada-defence-policy/docs/canada-defence-policy-report.pdf (accessed 18 April 2021), 49–57.

4 Ryan Dean, P. Whitney Lackenbauer, and Adam Lajeunesse, “Canadian Arctic defence policy: A synthesis of key documents, 1970–2013,” *Documents on Canadian Arctic Sovereignty and Security*, 2014, http://pubs.aina.ucalgary.ca/dcass/82112.pdf (accessed 29 September 2020); Shelagh Grant, *Sovereignty or Security?: Government Policy in the Canadian North, 1936–1950* (Vancouver: UBC Press, 1988), 3–4; see Nathaniel French Caldwell, Jr., *Arctic Leverage: Canadian Sovereignty and Security* (New York: Praeger Publishers, 1990).

5 David Lyon, Keven D. Haggerty, and Kirstie Ball, “Introducing surveillance studies,” in Kirstie Ball, Kevin Haggerty, and David Lyon, eds., *Routledge Handbook of Surveillance Studies* (New York: Routledge, 2012), 1.

6 Department of Defense, “DOD Dictionary of Military and Associated Terms,” January 2021, https://www.jcs.mil/Portals/36/Documents/Doctrine/pubs/dictionary.pdf (retrieved 1 June 2021), 206.

7 Canadian Armed Forces, *Arctic Integrating Concept,* Government of Canada (print, 2010), 30.

8 Michael Byers and Nicole Covey argue that dual-use search and rescue (SAR) assets support the bulk of Canada’s Arctic security requirements without contributing to Russian insecurity in the region, which could lead to instability through a classic security dilemma scenario. See Byers and Covey, ‘Arctic SAR and the “security dilemma,”’ *International Journal* 4, no. 74 (2019): 499–517; See also Kristian Åtland, “Interstate relations in the Arctic: An emerging security dilemma?” *Comparative Strategy* 2, no. 33 (2014): 145–166; Marzia Scopelliti and Elena Conde Pérez, “Defining security in a changing Arctic: Helping to prevent an Arctic security dilemma,” *Polar Record* 6, no. 52 (2016): 672–679.

9 For example, Kari Roberts, “Jets, flags, and a new Cold War? Demystifying Russia’s Arctic intentions,” *International Journal* 4, no. 65 (2010): 957–976; Jørgen Staun, “Russia’s strategy in the Arctic: Cooperation, not confrontation,” *Polar Record* 3, no. 53 (2017): 314–332.

10 Klaus Dodds, “We are a northern country: Stephen Harper and the Canadian Arctic,” *The Polar Record* 47, no. 4 (2011): 371–374; Klaus Dodds, “Graduated and paternal sovereignty: Stephen Harper, Operation Nanook 10, and the Canadian Arctic,” *Environment and Planning D: Society and Space* 30, no. 6 (2012): 989–1010.

11 James Manicom, “The domestic politics of disputed Arctic boundaries: The Canadian case,” *The Polar Record* 50, no. 2 (2014): 165–175.

12 P. Whitney Lackenbauer, “Arctic defence and security: Transitioning to the Trudeau government,” in P. Whitney Lackenbauer and Heather Nicol, eds., *Whole of Government Through an Arctic Lens* (Antigonish, NS: Mulroney Institute of Government, St. Francis Xavier University, 2017), 309; see Petra Dolata, “A new Canada in the Arctic? Arctic policies under Harper,” *Canadian Studies,* 78 (2015): 131–154.

13 Hannes Gerhardt, “Imagining the Arctic,” in Philip E. Steinberg and Jeremy Tasch, eds., *Contesting the Arctic: Politics and Imaginaries in the Circumpolar North* (I.B. Tauris, 2015), 2–6; see Elana Wilson Rowe, “Analyzing frenemies: An Arctic repertoire of cooperation and rivalry,” *Political Geography* 76 (January 2020): 102072; Staun, “Russia’s strategy in the Arctic,” 314–332; Jon Rahbek-Clemmensen, “The Ukraine crisis moves North: Is Arctic conflict spill-over driven by material interests?” *Polar Record* 1, no. 53 (2017): 1–15; Kari Roberts, “Understanding Russia’s security priorities in the Arctic: Why Canada-Russia cooperation is still possible,” *Canadian Foreign Policy Journal* 27, no. 2 (2020): 1–17; Michael Byers, “Crises and international cooperation: An Arctic case study,” *International relations* 31, no. 4 (2017): 375–402; Michael Byers, “Cold, dark, and dangerous: International cooperation in the Arctic and space,” *Polar Record* 1, no. 55 (2019): 32–47; see Valery Konyshev, Alexander Sergunin, and Sergei Subbotin, “Russia’s Arctic strategies in the context of the Ukrainian crisis,” *The Polar Journal* 1, no. 7 (2017): 104–124 for a discussion on the role of “spill-over” from the Ukraine crisis and Russian aggression into Arctic relations.

14 See Dean, Lackenbauer, and Lajeunesse, “Canadian Arctic defence policy,” for an overview of these shifts.

15 Roland Paris, “Are Canadians still liberal internationalists? Foreign policy and public opinion in the Harper era,” *International Journal* 69, no. 3 (2014): 274–307; see Thomas F. Keating, *Canada and world order: The Multilateralist Tradition in Canadian Foreign Policy* (Oxford: Oxford University Press, 2002).

16 Rob Huebert, “Understanding Arctic security: A defence of traditional security analysis,” in Wilfrid Greaves and P. Whitney Lackenbauer, eds., *Breaking Through: Understanding Sovereignty and Security in the Circumpolar Arctic* (Toronto: University of Toronto Press, 2021), 90.

17 Lackenbauer, “Arctic defence and security,” in Lackenbauer and Nicol, *Whole of Government Through an Arctic Lens*, 308–340.

18 For example, Dean, Lackenbauer, and Lajeunesse, “Canadian Arctic defence policy,” 1 and 12; see Adam Lajeunesse, “The Canadian Armed Forces in the Arctic: Purpose, capabilities, and requirements,” Canadian Defence and Foreign Affairs Institute, May 2015, https://www.policyschool.ca/wp-content/uploads/2019/06/FR-Canadian-Armed-Forces-Arctic-Lajeunesse.pdf (accessed 1 October 2020).

19 For a discussion of the DEW line in the context of Canada-US relations, see Adam Lajeunesse, “The Distant Early Warning Line and the Canadian battle for public perception,” *Canadian Military Journal* 8, no. 2 (2007): 51–59; and Joseph Jockel, *No Boundaries Upstairs: Canada, the United States, and the Origins of North American Air Defence, 1945–1958* (Vancouver: UBC Press, 1987). See also F. Robert Naka and William W. Ward, “Distant Early Warning Line radars: The quest for automatic signal detection,” *Lincoln Laboratory Journal* 2, no. 12 (2000): 181–204, for a historical look at the rapid technological development undertaken to meet the DEW line’s surveillance and warning needs regarding their technical requirements.

20 Rolf Tamnes and Kristine Offerdal, “Introduction,” in Tamnes and Offerdal, *Geopolitics and Security in the Arctic*, 28.

21 For an overview of surveillance efforts during the Cold War, see Adam Lajeunesse and Bill Carruthers, “The ice has ears,” *Canadian Naval Review* 3, no. 9 (2013): 1–9; Gary E. Weir, “The American Sound Surveillance System: Using the ocean to hunt soviet submarines, 1950–1961,” *International Journal of Naval History* 5, no. 2 (2006), https://www.ijnhonline.org/wp-content/uploads/2012/01/article_weir_aug06.pdf (accessed 14 April 2021); Dean, Lackenbauer, and Lajeunesse, “Canadian Arctic defence policy,” 9; and H.I. Sutton, “Canada’s secret Cold War submarine drone is still relevant today,” *Forbes*, 30 December 2020, https://www.forbes.com/sites/hisutton/2020/07/12/canada-secret-cold-war-drone-submarine-is-still-relevant-today/?sh=784429c71e96 (accessed 14 April 2021).

22 L.P. Dittmann, “In defence of defence: Canadian Arctic sovereignty and security,” *Journal of Military and Strategic Studies* 11, no. 3 (2009): 53.

23 William Carruthers, “An array of blunders: The Northern Watch Technology Demonstration Project,” *Papers in Northern & Maritime Security*, Mulroney Institute of Government*,* St. Francis Xavier University, 2018, https://cdainstitute.ca/wp-content/uploads/2018/06/An-Array-of-Blunders.pdf (accessed 8 January 2021); Garry J. Heard, “Canadian Arctic Underwater Sentinel Experiment (CAUSE)—Gascoyne Inlet Camp (GIC) 2017 field trial,” Defence Research and Development Canada, May 2018, https://cradpdf.drdc-rddc.gc.ca/PDFS/unc304/p806471_A1b.pdf (accessed 14 April 2021), 4; P. Whitney Lackenbauer and Adam Lajeunesse, “The Canadian Armed Forces in the Arctic: Building appropriate capabilities,” *Journal of Military and Strategic Studies* 16, no. 4 (2016): 34–35, 53.

24 Several factors make a WoG approach difficult in practice, including institutional asymmetry, differences in departmental culture, and siloed bureaucracies. See Karen Everett and Emily Yamashita, “Whole of Government in the Canadian Arctic,” in Lackenbauer and Nicol, *Whole of Government Through an Arctic Lens*, 295–307.

25 P. Whitney Lackenbauer, “Tolerant allies: The Joint Arctic Weather Stations, Canadianization, and Canada-United States relations in the Cold War Arctic,” *International Journal* 4, no. 75 (2020): 487–502; See also Daniel Heidt, “Clenched in the JAWS of America? Canadian sovereignty and the joint Arctic weather stations, 1946–1972,” in P. Whitney Lackenbauer, ed., *Canadian Arctic Sovereignty and Security: Historical Perspectives* (Calgary: *The Calgary Papers in Military and Strategic Studies* 4, 2011), 145–170; Gordon W. Smith, “Weather stations in the Canadian North and sovereignty,” *Journal of Military and Strategic Studies* 3, no. 11 (2009): 1–63.

26 Jackie Dawson et al., “Navigating weather, water, ice and climate information for safe polar mobilities,” World Weather Research Programme*,* World Meteorological Foundation, 2017, https://www.polarprediction.net/fileadmin/user_upload/www.polarprediction.net/Home/Organization/Task_Teams/PPP-SERA/WWRP_PPP_No_5_2017_11_OCT.pdf (accessed 17 April 2021); the YOPP data set is identified by the DND within cooperation efforts under the International Cooperative Engagement Program for Polar Research (ICE-PPR) composed of member nations’ defence departments and other government agencies engaged in polar research. See Dale F. Reading, “International Cooperative Engagement Program for Polar Research (ICE-PPR),” National Defence (28 February–1 March 2017), https://asp-net.org/sites/default/files/website_files/ICE%20PPR2.pdf (accessed 14 April 2021).

27 Government of Canada, “Arctic and Northern Policy Framework: Safety, security, and defence chapter.”

28 See David H. Boteler, “Dealing with space weather: The Canadian experience,” in Natalia Buzulukova, ed., *Extreme Events in Geospace: Origins, Predictability, and Consequence* (Amsterdam: Elsevier, 2018), 645–656; Patrick Perron, “Space weather situational awareness and its effects upon a joint, interagency, domestic, and Arctic environment,” *Canadian Military Journal* 14, no. 4 (2014): 18–27.

29 Torbjørn Pedersen, “Polar research and the secrets of the Arctic,” *Arctic Review on Law and Politics* 10 (2019): 103–129.

30 For example, the Nahendeh Kehotsendi project in Hay River, NWT, led by the Kátł'odeeche First Nation, “establishes a land monitoring project based on traditional knowledge and experience. However, it uses contemporary technology and techniques as required.” See Government of Canada, “Indigenous guardians pilot program map,” Year 3, 10 December 2020, https://www.canada.ca/en/environment-climate-change/services/environmental-funding/indigenous-guardians-pilot/map.html (accessed 17 April 2021).

31 Transport Canada, “Oceans Protection Plan,” Government of Canada, 8 July 2020, https://tc.canada.ca/en/initiatives/oceans-protection-plan (accessed 17 April 2021).

32 See Government of Canada, “Frequently asked questions – RADARSAT Constellation Mission (RCM),” 16 March 2021, https://www.asc-csa.gc.ca/eng/satellites/radarsat/faq.asp (accessed 18 April 2021).

33 Government of Canada, *Marine Identification Systems*, 26 July 2019, https://www.ccg-gcc.gc.ca/maritime-security-surete-maritime/systeme-identification-system-eng.html (accessed 17 April 2021); Government of Canada, *Maritime Security Contributions*, 26 July 2019, https://www.ccg-gcc.gc.ca/publications/maritime-security-surete-maritime/contributions/index-eng.html (accessed 17 April 2021).

34 P. Whitney Lackenbauer, *The Canadian Rangers: A Living History* (Vancouver: UBC Press, 2013), 259.

35 Government of Canada, *Strong, Secure, Engaged*, 80.

36 P. Whitney Lackenbauer and Peter Kikkert, “‘An important international crossroads’: Implementing Canada’s Arctic priorities in Strong, Secure, Engaged,” Report for symposium hosted by the Centre for National Security Studies, Canadian Forces College, Ottawa, ON, https://www.cfc.forces.gc.ca/CNSS/arctic-eng.pdf?cfc (accessed 14 April 2021), 22; Government of Canada, “Canadian Rangers,” 11 March 2021, http://www.army-armee.forces.gc.ca/en/canadian-rangers/index.page (accessed 17 June 2021).

37 Defence Research and Development Canada, “All Domain Situational Awareness Science and Technology Program,” Government of Canada, 12 February 2018, https://www.canada.ca/en/defence-research-development/programs/all-domain-situational-awareness-program.html (accessed 2 October 2020).

38 *Ibid*.

39 Public Works and Government Services Canada, “Defence Research and Development Canada (DRDC) Innovation Call for Proposals (CFP),” Government of Canada, n.d., https://buyandsell.gc.ca/cds/public/2016/12/05/b4d6888aa61d3d7a9ec59a370dfdabe1/drdc_innovation_cfp.pdf (accessed 3 October 2020), 33, 35.

40 Fergusson argues that the NWS’s replacement will require multi-domain surveillance through an integrated sensor system to defend against future threats. See James Fergusson, “Missed opportunities: Why Canada’s North Warning System is overdue for an overhaul,” Macdonald-Laurier Institute, January 2020, https://macdonaldlaurier.ca/files/pdf/20191219_NORAD_Fergusson_COMMENTARY_FWeb.pdf (accessed 18 April 2021), 3.

41 National Defence, “Quantum S&T Strategy: Preparing for technological disruptions in the future operating environment,” Government of Canada, 2021, https://www.canada.ca/content/dam/dnd-mdn/documents/reports/DGM-61120-DD8_DNDCAF_QuantumSTStrategy_EN_v3.pdf (accessed 18 April 2021).

42 National Defence, “Government of Canada announces contract award to the University of Waterloo for research and development in support of Arctic surveillance,” Government of Canada, 12 April 2018, https://www.canada.ca/en/department-national-defence/news/2018/04/government-of-canada-announces-contract-award-to-the-university-of-waterloo-for-research-and-development-in-support-of-arctic-surveillance0.html (accessed 18 April 2021).

43 For a more detailed history, see “A brief history of NORAD,” Office of the Command Historian, 13 May 2016, https://www.norad.mil/Portals/29/Documents/History/A%20Brief%20History%20of%20NORAD_May2016.pdf?ver=2016-07-07-114925-133 (accessed 2 June 2021).

44 NORAD and USNORTHCOM are separate commands but share complementary defensive missions. See North American Aerospace Defense Command, http://www.norad.mil/ (accessed 1 June 2021); and NORAD and USNORTHCOM, “Command narrative,” 27 February 2017, https://www.northcom.mil/Portals/28/Command%20Narrative_27%20Feb%2017.pdf?ver=2018-01-19-120844-210#:∼:text=NORAD%20and%20USNORTHCOM%20must%20partner,both%20the%20U.S.%20and%20Canada.&text=Our%20other%20USNORTHCOM%20regional%20partners,illicit%20trafficking%20and%20criminal%20violence (accessed 4 June 2021).

45 Department of Defense, “Report to Congress: Department of Defense Arctic Strategy,” Office of the Under Secretary of Defense for Policy, June 2019, https://media.defense.gov/2019/Jun/06/2002141657/-1/-1/1/2019-DOD-ARCTIC-STRATEGY.PDF (accessed 3 June 2021).

46 Terrence J. O’Shaughnessy and Peter M. Fesler, “Hardening the Shield: A credible deterrent & capable defense for North America,” Canada Institute and Wilson Center, September 2020, https://www.wilsoncenter.org/sites/default/files/media/uploads/documents/Hardening%20the%20Shield_A%20Credible%20Deterrent%20%26%20Capable%20Defense%20for%20North%20America_EN.pdf (accessed 1 June 2021); “NORAD and USNORTHCOM Strategy: Executive Summary,” March 2021, https://www.northcom.mil/Portals/28/(U)%20NORAD-USNORTHCOM%20Strategy%20EXSUM%20-%20Signed.pdf (accessed 1 June 2021); see Ryan Dean and Nancy Teeple, “Introduction: The missing chapter from Strong, Secure, Engaged,” in R. Dean and N. Teeple, eds., *Shielding North America: Canada’s Role in NORAD Modernization* (NAADSN Engage Series, 2021), xii, https://www.naadsn.ca/wp-content/uploads/2021/03/NAADSN-engage4-NORAD-NT-RD-upload.pdf (accessed 15 April 2021).

47 See Department of Defense, “Summary of the 2018 National Defense Strategy of the United States of America,” 2018, https://dod.defense.gov/Portals/1/Documents/pubs/2018-National-Defense-Strategy-Summary.pdf (accessed 21 April 2021).

48 Dean and Teeple, “Introduction,” xii. Formally, this is termed as “Joint All Domain Command and Control” (JADC2). See “Joint All-Domain Command and Control (JADC2),” 18 March 2021, Congressional Research Service, https://fas.org/sgp/crs/natsec/IF11493.pdf (accessed 2 June 2021).

49 O’Shaughnessy and Fesler, “Hardening the Shield,” 10.

50 “NORAD and USNORTHCOM Strategy,” 6.

51 Rob Huebert, “The Arctic and strategic defence of North America: Revision of the long ‘polar watch,’” in Christian Leuprecht, Joel J. Sokolsky, and Thomas Hughes, eds., *North American Strategic Defense in the 21st Century: Security and Sovereignty in an Uncertain World* (SpringerLink, 2018), 176; see “NORAD and USNORTHCOM Strategy,” 3.

52 Theresa Hitchens, “The key to all-domain warfare is ‘predictive analysis:’ Gen. O’Shaughnessy,” *Breaking Defense*, 5 May 2020, https://breakingdefense.com/2020/05/the-key-to-all-domain-warfare-is-predictive-analysis-gen-oshaughnessy/ (accessed 15 April 2021).

53 Another major application for AI in surveillance technology is through automation in navigation and detection systems in vehicle platforms, such as aerial and underwater unmanned vehicles (UUV) for Arctic ISR. For example, see the Canadian Pathfinder project with Transport Canada: Kelsey Reichmann, “Iris Automation launches Canadian Pathfinder Project to streamline unmanned BVLOS approvals,” *Aviation Today*, 17 February 2021, https://www.aviationtoday.com/2021/02/17/iris-automation-launches-canadian-pathfinder-project-streamline-unmanned-bvlos-approvals/ (accessed 5 June 2021); and Cellula Robotics’ Solus-LR project under the ADSA program: “Solus-LR successfully completes first sea trials,” Cellula Robotics, 20 December 2019, https://www.cellula.com/news?year=2019 (accessed 5 June 2021).

54 O’Shaughnessy and Fesler, “Hardening the Shield,” 9; “NORAD and USNORTHCOM Strategy,” 7.

55 For example, the Fusion of Open Data Sources with Automated Identification System (AIS) and Earth Observation Data for Enhanced Marine Domain Awareness (MDA) project and the Compression of the Tasking, Collection, Processing, Exploitation and Dissemination (TCPED) Cycle project being pursued under the ADSA program. These projects are concerned with using satellites like RCM to create accurate and uninterrupted pictures of Canada’s Arctic territory. See Government of Canada, “All Domain Situational Awareness Program,” Projects.

56 Ibid.

57 Andrea Charron, “Responding to ‘Hardening the Shield: A credible deterrent & capable defense for North America,’” in Teeple and Dean, *Shielding North America*, 85.

58 Ibid., 87; Ryan Dean and Nancy Teeple, “First report: Awareness and sensors,” *CDA Institute NORAD Modernization Forum*, https://cdainstitute.ca/wp-content/uploads/2020/09/CDAI-NORAD-SENSORS-REPORT-1-3.pdf (accessed 15 April 2021), 6.

59 Charron, “Responding to ‘Hardening the Shield,’” 88.

60 Frank L. Smith III, “Quantum technology hype and national security,” *Security Dialogue* 5, no. 51 (2020): 500.

61 For instance, the ADSA program has a total budget of $133 million, relatively moderate by defence standards.

62 Nancy Teeple, “The future of Canadian participation in missile defence,” in Teeple and Dean, *Shielding North America*, 128. See 126–130 for a discussion on options for Canada’s participation in missile defence.

63 Year of Polar Prediction, *YOPP data set, Observation Layer for the Arctic* (5 June 2020), https://www.polarprediction.net/data/yopp-observations-layer/ (accessed 5 June 2020).

64 Canadian High Arctic Ionospheric Network (CHAIN), *Real-Time Scintillation Map*, http://chain.physics.unb.ca/chain/ (accessed 15 May 2020).

65 Government of Canada, *RADARSAT Mosaics*, 18 September 2020, https://www.canada.ca/en/environment-climate-change/services/ice-forecasts-observations/latest-conditions/products-guides/satellite-images-mosaics-animations.html (accessed 14 April 2021).

